# Computational Foretelling and Experimental Implementation of the Performance of Polyacrylic Acid and Polyacrylamide Polymers as Eco-Friendly Corrosion Inhibitors for Copper in Nitric Acid

**DOI:** 10.3390/polym14224802

**Published:** 2022-11-08

**Authors:** Arafat Toghan, Ahmed Fawzy, Areej Al Bahir, Nada Alqarni, Moustafa M. S. Sanad, Mohamed Khairy, Abbas I. Alakhras, Ahmed A. Farag

**Affiliations:** 1 Chemistry Department, College of Science, Imam Mohammad Ibn Saud Islamic University (IMSIU), Riyadh 11623, Saudi Arabia; 2 Chemistry Department, Faculty of Science, South Valley University, Qena 83523, Egypt; 3 Chemistry Department, Faculty of Applied Sciences, Umm Al-Qura University, Makkah 21955, Saudi Arabia; 4 Chemistry Department, Faculty of Science, Assiut University, Assiut 71516, Egypt; 5 Chemistry Department, Faculty of Science, King Khalid University, Abha 64734, Saudi Arabia; 6 Department of Chemistry, College of Sciences and Arts in Balgarn, University of Bisha, Bisha 61922, Saudi Arabia; 7 Central Metallurgical Research & Development Institute, P.O. Box 87, Helwan, Cairo 11421, Egypt; 8 Chemistry Department, Faculty of Science, Benha University, Benha 13518, Egypt; 9 Egyptian Petroleum Research Institute (EPRI), Cairo 11727, Egypt

**Keywords:** copper, polyelectrolytes, corrosion inhibitors, mechanisms, thermodynamic, kinetics, theoretical investigation

## Abstract

Copper is primarily used in many industrial processes, but like many other metals, it suffers from corrosion damage. Polymers are not only one of the effective corrosion inhibitors but also are environmentally friendly agents in doing so. Hence, in this paper, the efficacy of two polyelectrolyte polymers, namely poly(acrylic acid) (PAA) and polyacrylamide (PAM), as corrosion inhibitors for copper in molar nitric acid medium was explored. Chemical, electrochemical, and microscopic tools were employed in this investigation. The weight-loss study revealed that the computed inhibition efficiencies (% IEs) of both PAA and PAM increased with their concentrations but diminished with increasing HNO_3_ concentration and temperature. The results revealed that, at similar concentrations, the values of % IEs of PAM are slightly higher than those recorded for PAA, where these values at 298 K reached 88% and 84% in the presence of a 250 mg/L of PAM and PAA, respectively. The prominent IE% values for the tested polymers are due to their strong adsorption on the Cu surface and follow the Langmuir adsorption isoform. Thermodynamic and kinetic parameters were also calculated and discussed. The kinetics of corrosion inhibition by PAA and PAM showed a negative first-order process. The results showed also that the used polymers played as mixed-kind inhibitors with anodic priority. The mechanisms of copper corrosion in nitric acid medium and its inhibition by the tested polymers were discussed. DFT calculations and molecular dynamic (MD) modelling were used to investigate the effect of PAA and PAM molecular configuration on their anti-corrosion behavior. The results indicated that the experimental and computational study are highly consistent.

## 1. Introduction

Corrosion of metals, a natural phenomenon, is the gradual destruction of metals and alloys by chemical and/or electrochemical reactions with their environments as well as degradation of their useful properties and structures, including strength and appearance. This natural phenomenon costs the world’s economy much money each year. Such a phenomenon not only raises economic concerns but also causes serious environmental impact. Corrosion inhibitors are materials employed to protect or inhibit the corrosion phenomenon of metallic surfaces [1,2,3,4]. The proficiencies of the inhibitors were set to be dependent on their abilities to adsorb on the metallic surfaces [5,6,7,8,9,10,11]. These inhibitors are organic compounds comprising functional groups, namely aromatic and/or heterocyclic rings, heteroatoms such as oxygen, nitrogen, sulfur, and π-electrons, in their structures, which can aid their adsorption onto the metal surfaces [12,13,14].

Various applications of polymers in many stabilizations flocculation processes have resulted in a wide examination of the adsorption mechanism of polymeric substances at the solid/solution interface [15,16,17]. In metallic corrosion inhibition, they represent a set of chemically stable, biodegradable, and ecofriendly macromolecules with unique inhibiting strengths for metal protection [18]. The inhibition potentials of polymers were explained on the basis of their macromolecular weights, chemical structures, and the nature of the metallic surface. Polymers have been widely employed as metallic corrosion inhibitors because of their capabilities to complex with the metallic surfaces due to presence of lone pairs of electrons (as well as pi electrons) on the polymeric molecules. The formed complexes effectively blanket the metallic surfaces from the attack of the corrosive environment [19,20]. Because of their stability in acidic media, polymers have been widely employed as corrosion inhibitors for various metals and alloys in different aggressive acidic media [6,7,8,15,18,19,20,21,22,23,24,25,26,27,28,29,30,31].

Poly(acrylic acid) (PAA) is a synthetic, non-toxic, and water-soluble anionic polyelectrolyte. Its acid base and water-attracting properties are the bases of various industrial applications [32]. Poly(acrylic acid) and its derivatives have medical applications and have been used in paints and cosmetics. PAA films can be deposited on surfaces to protect them from corrosion. Polyacrylamide (PAM) is a polyolefin that has been greatly inspected regarding the environment and human health [31]. It is highly water-absorbent, forming a soft gel when hydrated. It is used in the oil and mineral industries. It is applied in water treatment because it can flocculate solids in a liquid. PAM is also used in molecular biology applications. The two mentioned polymeric compounds (PAA and PAM) contain heteroatoms such as oxygen atoms in PAA and oxygen and nitrogen atoms in PAM, which can form complexes with the metal surface through lone pairs of electrons. These complexes with a polymeric nature form adherent protective layer(s) on the metal surface, which acts as a barrier to aggressive environments. Therefore, various corrosion inhibition works using poly(acrylic acid) have been performed, such as corrosion inhibition of iron in sulfuric acid solution [24] and aluminum in weakly alkaline solutions [22]. Furthermore, polyacrylamide was examined as an inhibitor for the corrosion of iron [26,27] and mild steel [28] in sulfuric acid solutions, iron [29] and aluminum [30] in HCl solutions, and C-steel in ground water [31].

Copper is a strategic metal in various industrial fields, such as in the car industry, oil refineries, sugar factories, power stations, heat exchangers, cooling towers, etc. [30,31,32,33,34,35]. This is because of some favorable properties such as good corrosion resistance, high electrical and thermal conductivity, good mechanical workability, as well as its comparatively low cost [36,37,38]. In addition, copper alloys are regarded as the chief ingredients in technological device utilizations, such as in the manufacture of wire and sheets in electronics [39]. However, copper is susceptible to corrosion phenomenon in aggressive media [40,41,42,43]. The corrosion of copper and formation of corrosion products on its surface due to its expose to the aggressive environments have a negative effect on the performance of metallic systems constructed from copper and may reduce its efficiency [44,45,46]. The possibility of passive film formation on copper surface is low in highly aggressive environments [40,41]. According to widespread use of copper in different industries, the issue of corrosion and its protection has attracted a great deal of attention, and many studies have been conducted to date [33,34,35,36,47,48]. One of the aggressive media for copper is HNO_3_ media, and thus, the protection of copper from corrosion attacks in these media is important [49,50]. The corrosion of copper in nitric acid media results in generation of Cu^2+^ ions, which transfer from the copper surface into the medium.

**Figure 1:**
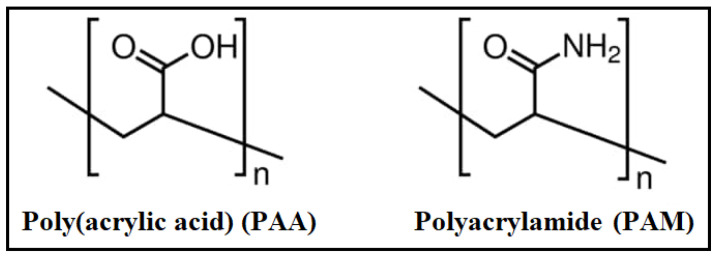
Structures of the two examined eco-friendly polymers.

The present study aims to evaluate the corrosion inhibition aspects of poly(acrylic acid) and polyacrylamide polymers (see Figure 1) for the first time for copper in 1.0 M HNO_3_ medium by means of weight loss (WL), potentiodynamic polarization (PDP), electrochemical impedance spectroscopy (EIS), and scanning electron microscopy (SEM) techniques. Moreover, thermodynamic, kinetics, and mechanistic features were investigated. DFT calculations and molecular dynamic (MD) modelling were used to investigate the effect of PAA and PAM molecular configuration on their anti-corrosion behavior.

## 2. Experimental

### 2.1. Materials

Most chemicals utilized in this investigation were from Sigma-Aldrich. The solutions were made using bi-distilled water. The main corrosive medium used in all investigations was 1.0 M HNO3, which was prepared by diluting analytical-grade 70% HNO_3_ in bi-distilled water, and the acid concentration was standardized using NaOH solution. The poly(acrylic acid) (PAA) and polyacrylamide (PAM) with molecular weight of 45,000 and 40,000 g/mol, respectively, were used as corrosion inhibitors. Solutions of poly(acrylic acid) and polyacrylamide were utilized in a range of concentrations from 50 to 250 mg/L. Experiments were carried out on copper specimens (Merck) with the following composition (wt.%): 0.030 Fe, 0.021 Pb, 0.011 Ni, 0.005 Si, and the remainder Cu. The temperature was adjusted to 298 K using a thermostat. Every experimental run was repeated about three times under similar circumstances to test the reproducibility.

### 2.2. Techniques

This investigation was performed using chemical (weight loss (WL)), electrochemical (potentiodynamic polarization (PDP) and electrochemical impedance spectroscopy (EIS)), and microscopic (scanning electron microscopy (SEM)) techniques. WL experiments were carried out in temperature-controlled vessels. Copper specimens were sheets with areas around 12 cm^3^. Before each experiment, the surfaces of copper sheets were mechanically polished with variety of emery paper up to a grade 1500 and degreased with acetone and bi-distilled water. Then, the cleaned sheet was immersed in 100 mL of the test solution for a period of time up to 4 h. The electrochemical experiments (PDP and EIS) were recorded using PGSTAT30 potentiostat/galvanostat with a temperature controller. Three compartment cells, namely copper (working electrode), Pt sheet (counter electrode), and saturated calomel (reference electrode), were used. Prior to each experiment, the utilized cell and the working electrode were prepared as mentioned in our earlier studies [11,12,13]. Then, the copper electrode was immersed in the corrosive medium (1.0 M HNO_3_ medium) without and with the tested polymers until a fixed potential was attained at OCP (open-circuit potential). PDP curves were recorded in the potential range of ±250 mV at a scan rate of 2.0 mV s^−1^. The measurements of EIS studies were carried out in the frequency range of 100 kHz to 0.1 Hz and the amplitude of 5.0 mV (peak to peak) using AC signals at OCP. Surface examination of copper sheets was explored before and after adding 250 mg/L of the tested polymers to the corrosive medium for about 12 h using SEM. The imaging was done using JEOL Scanning electron microscope (SEM), model T-200, with a repetition voltage of 10.0 kV. After this, the copper sheets were removed from the test medium, dried, and submitted for the SEM analysis.

### 2.3. Theoretical Studies

The Gaussian-9 software was used to obtain the data on the regular optimization assembly and electron mass spreading of the inhibitor molecules. Theoretical computations were carried out utilizing atomic orbitals as the basis set using density functional theory (DFT) at B3LYP and 6–31*G (d,p) technique. Some essential parameters were acquired from theoretical calculations to further describe the inhibitions property as the energy of the Frontier (*E*_HOMO_ and *E*_LUMO_) and the gap energy (Δ*E* = *E*_LUMO_ – *E*_HOMO_). Material Studio 2017 software (Accelrys Inc.) was used for the MD simulations, which were mostly constructed on the Forcite module. Molecules of inhibitors have been tested in a simulated box with periodic border settings, which ensured that an illustrative portion for restrictive substrate was free of random border impact. Copper atom (111) was sliced lengthwise its level in a 5Å sheet and extended into a (ten × ten) supercell to confirm a large, sufficient surface for the molecule’s communication. In addition, the H_2_O molecules were 175 and 1 molecule of the investigated inhibitors.

## 3. Results and Discussion

### 3.1. WL Measurements

#### 3.1.1. Effect of Polymers’ Concentrations

**Figure 2:**
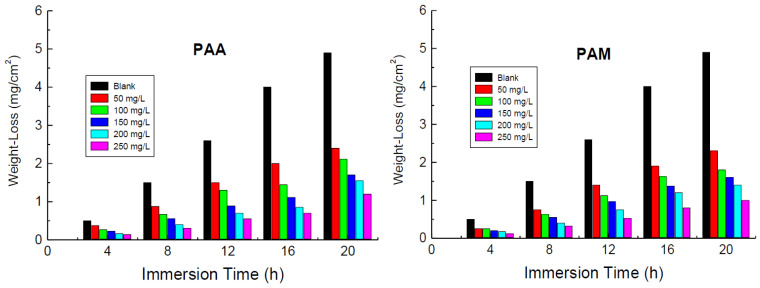
Weight-loss versus immersion time for the corrosion of copper in 1.0 M HNO_3_ medium and with addition of poly(acrylic acid) (PAA) and polyacrylamide (PAM) at 298 K.

WL measurements for copper were performed in a certain concentration of the corrosive medium (blank), viz., 1.0 M HNO_3_ medium, without and with numerous concentrations (50–250 mg/L) of the tested polymers (poly(acrylic acid) and polyacrylamide) at various temperatures, namely 288, 298, 308, and 318 K. Figure 2 shows only the WL measurements at 298 K as a representative example. The corrosion rates (*CR* in mils penetration per year) were calculated from Equation (1) [51]:

(1) CR=KW/Atd

where *K* is a constant (3.45 × 10^6^), *W* is the WL in grams, *A* is copper sheet area in cm^2^, *t* is the time in hours, and *d* is the copper density. The values of the inhibition efficiencies (% IE) and the degree of surface coverage (*θ*) of the tested polymers were computed from Equation (2) [52]:

(2) $$\%\;\mathrm{IE}\;=\left[1-\frac{CR_{inh}}{CR}\right]\times 100=\theta \times 100$$

where *CR* and *CR_inh_* are the corrosion rates without and with addition of the tested polymer (inhibitor), respectively.

**Figure 3:**
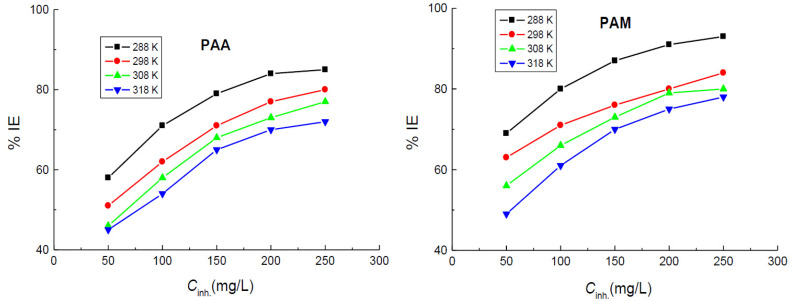
Dependence of the inhibition efficiencies (% IEs) of poly(acrylic acid) (PAA) and polyacrylamide (PAM) on their concentrations in the corrosion of copper in 1.0 M HNO_3_ medium at different temperatures.

The calculated values of *CR*, % IE, and *θ* at various temperatures are listed in Table 1. The results indicated that *CR* values were decreased, and the values of % IEs of the tested polymers were increased with the polymers’ concentrations, as illustrated in Figure 3. This behavior can be attributed to the augmented adsorption of the polymers’ molecules on the vacant sites on the copper surface, with the increase of their concentrations leading to reduced values of *CR* and enhanced %IE values. Hence, the tested polymers are regarded as efficient inhibitors for the corrosion of copper in 1.0 M nitric acid medium. Furthermore, the results revealed that, at similar concentrations, the values of % IEs of PAM are slightly higher than those recorded for PAA.

#### 3.1.2. Effect of Corrosive Medium Concentration

**Figure 4:**
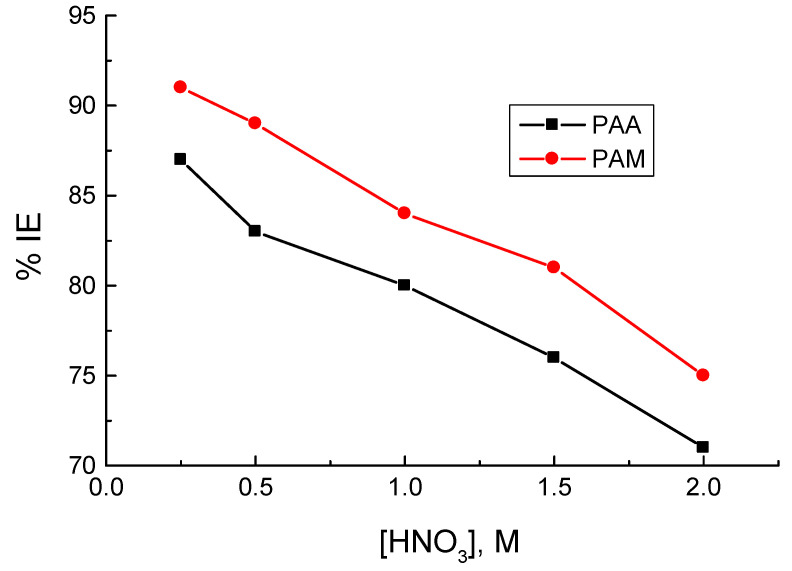
Dependence of the inhibition efficiencies (% IEs) of poly(acrylic acid) (PAA) and polyacrylamide (PAM) on the corrosive medium concentration in the corrosion of copper in HNO_3_ media at 298 K.

In order to illuminate the effect of the corrosive medium concentration on the % IEs of the tested polymers, WL measurements for copper were performed in various concentrations of the corrosive medium (0.25 to 2.0 M HNO_3_) in the presence of 250 mg/L of the tested polymers at 298 K, as illustrated in Figure 4. The figure signifies that increasing the concentration of the corrosive medium appreciably decreased the values of % IEs. This can be attributed to the augmented aggressiveness of the corrosive medium with concentration.

#### 3.1.3. Effect of Temperature

**Figure 5:**
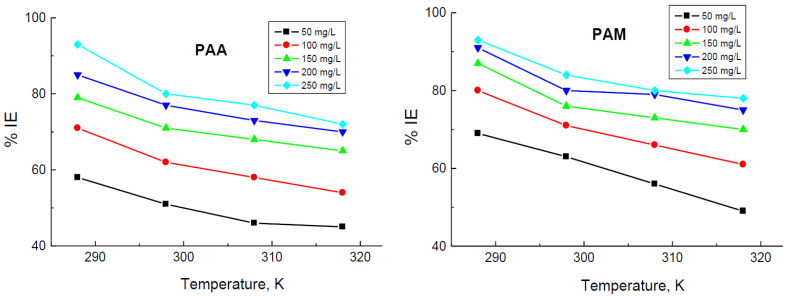
Dependence of the inhibition efficiencies (% IEs) of poly(acrylic acid) (PAA) and polyacrylamide (PAM) on temperature in the corrosion of copper in 1.0 M HNO_3_ medium.

In order to investigate the efficacies of the added polymers to the examined corrosive medium and to validate the stabilities of the adsorption layer(s) (protective film(s)), which was suggested to construct on the copper surface at higher temperatures as well as to evaluate the thermodynamic and activation parameters of the corrosion process, WL measurements were performed at diverse temperatures (288–318 K). The values of *CR* of copper and those of both % IEs and *θ* in the presence of different concentrations of the tested polymers were calculated and are listed in Table 2. The obtained results indicated that rising temperature led to an increase in the values of CRs in both the corrosive medium and in the presence of the tested polymers. Thus, the % IE values were diminished with temperature, as shown in Figure 5, indicating desorption of the polymeric molecules from the copper surface with temperature that supports physical adsorption of the tested polymers on the copper surface [53,54].

#### 3.1.4. Adsorption Consideration

**Figure 6:**
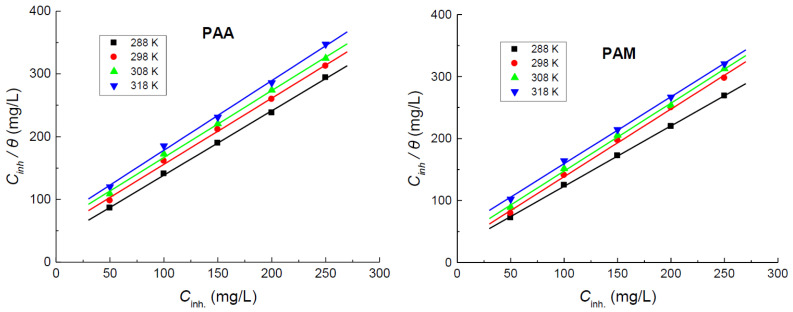
Langmuir adsorption isotherms for poly(acrylic acid) (PAA) and polyacrylamide (PAM) adsorbed on copper surface in 1.0 M HNO_3_ medium at different temperatures.

The present results indicated that the tested polymers were set to be efficient inhibitors against the corrosion of copper in 1.0 M HNO_3_ medium. This performance can be related to strong adsorption of the polymeric molecules on the copper surface to construct protective layer(s) [24,25,26,27,28,29]. To illuminate the adsorption mechanism that accord with the obtained results, the values of *θ* of the tested polymers at their applied concentrations were inserted in different adsorption isotherms as Langmuir, Frumkin, Temkin, Freundlich, etc. The acquired results at different temperatures were found to follow the Langmuir adsorption isotherm (Figure 6) defined by Equation (3) [55],

(3) $$\frac{C_{inh}}{\theta }=\frac{1}{K_{ads}}+C_{inh}$$

where *K_ads_* is the adsorption constant. Values of *K_ads_* were evaluated and are listed in Table 2.

#### 3.1.5. Thermodynamic Parameters

Thermodynamic parameters of the adsorption process, namely standard free energy ($\Delta G_{ads}^{o}$), standard heat ($\Delta H_{ads}^{o}$), and standard entropy $\Delta S_{ads}^{o}$), can provide considerable knowledge about the mechanism of corrosion inhibition. The values of $\Delta G_{ads}^{o}$ were determined at different temperatures using Equation (4) [56]:

(4) $$\Delta G_{ads}^{o}=-RTln\left(55.5k_{ads}\right)$$

The obtained higher negative values of $\Delta G_{ads}^{o}$ (inserted in Table 2) indicated the spontaneity of the adsorption process and stability of the adsorbed layer(s) on the copper surface. Additionally, these values illuminated that the nature of adsorption was mixed between physical and chemical adsorption [57]. The values of $\Delta H_{ads}^{o}$ were calculated using the Van’t Hoff equation as follows [58]:

(5) $$ln\left(k_{ads}\right)=\frac{-\Delta H_{ads}^{o}}{RT}+constant$$

**Figure 7:**
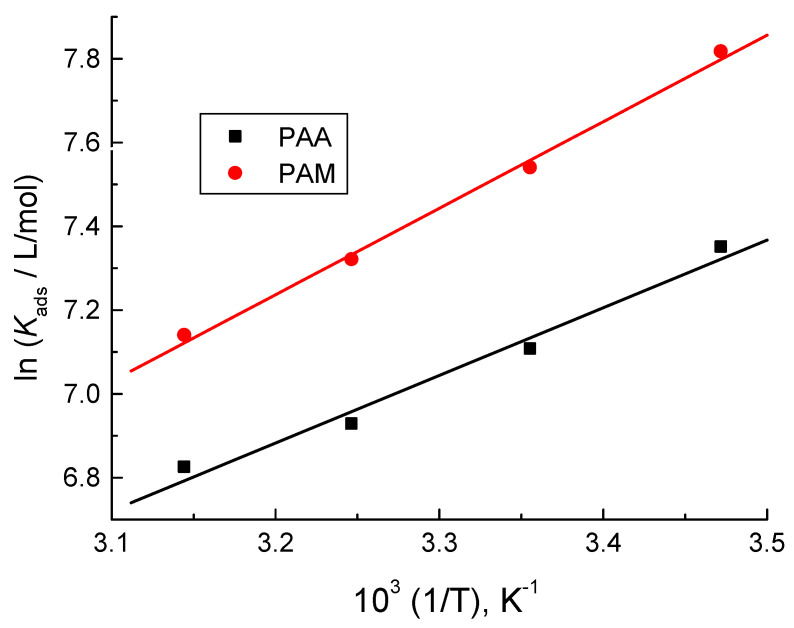
Van’t-Hoff plots for the tested poly(acrylic acid) (PAA) and polyacrylamide (PAM) adsorbed on the copper surface in 1.0 M HNO_3_ medium.

The plots of ln *K_ads_* vs. 1/*T* were found to be straight, as illustrated in Figure 7. The values of ∆*H*^o^*_ads_* were calculated from these plots and are inserted also in Table 2. The negative values of ∆*H*^o^*_ads_* accord with the exothermic physical adsorption [59].

The ∆*S*^o^*_ads_* values were calculated via Gibbs–Helmholtz equation, Equation (6):

(6) $$\Delta G_{ads}^{o}=\Delta H_{ads}^{o}-T\Delta S_{ads}^{o}$$

The gained positive values of $\Delta S_{ads}^{o}$ (Table 2) indicated an increase in the disorder of the polymers’ molecules during their adsorption on the copper surface [60].

#### 3.1.6. Kinetic Parameters

**Figure 8:**
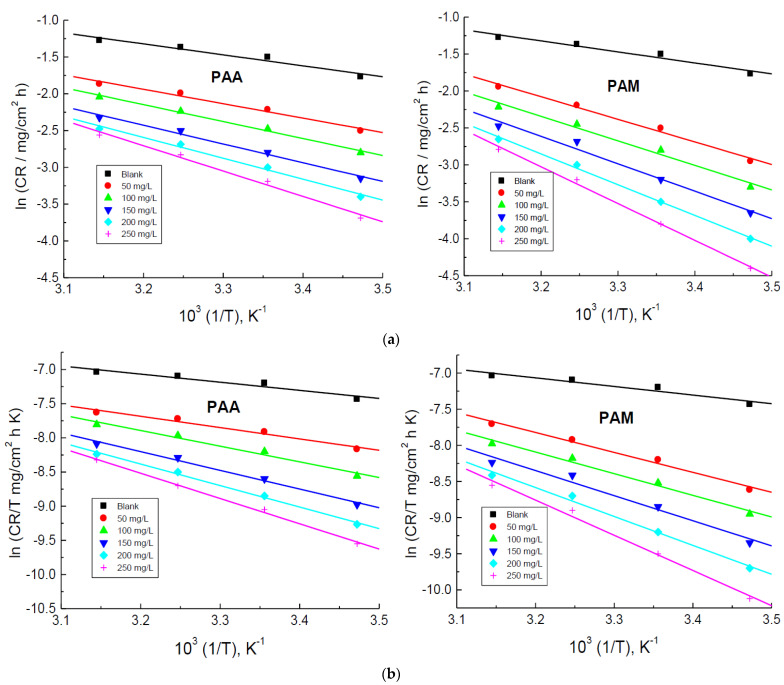
(**a**). Arrhenius plots for the corrosion of copper in 1.0 M HNO_3_ medium and with addition of poly(acrylic acid) (PAA) and polyacrylamide (PAM). (**b**). Transition state plots for the corrosion of copper in 1.0 M HNO_3_ medium and with addition of poly(acrylic acid) (PAA) and polyacrylamide (PAM).

The Arrhenius equation [61] describes the relationship between *CR* and temperature as follows:

(7) $$lnCR=lnA-\frac{E_{a}^{*}}{RT}$$

where $E_{a}^{*}$ is the activation energy. The Arrhenius plots shown in Figure 8a were set to be linear, and the values of $E_{a}^{*}$ were calculated and are listed in Table 3. The results indicated that the *E*_a_* values obtained in the presence of the tested polymers were found to be higher than that obtained in the corrosive medium itself. This behavior indicates strong adsorption of the polymeric molecules, leading to reduce the corrosion rates. In addition, the gained values of $E_{a}^{*}$ were set to be in the range of physical adsorption [62]. These outcomes are in agreement with the acquired values of both $\Delta G_{ads}^{o}$ and $\Delta H_{ads}^{o}$, indicating the validity of the present work.

The values of enthalpy of activation (ΔH*) and entropy of activation (ΔS*) for the corrosion process were evaluated the following equation [63]:

(8) $$ln\left(\frac{CR}{T}\right)=\left(ln\frac{R}{Nh}+\frac{\Delta S^{*}}{R}\right)-\frac{\Delta H^{*}}{R}\frac{1}{T}$$

where *N* is Avogadro’s number (6.02214076 × 10^23^), and *h* is Planck’s constant (6.626176 × 10^−34^ Js). The values of both ΔH* and ΔS* were evaluated from these plots (Figure 8b) and are listed in Table 3. The gained positive values of both ΔH* and ΔS* in the presence of the tested polymers suggested endothermic nature of corrosion inhibition and increase in the polymeric molecules disorder, respectively [64].

#### 3.1.7. Kinetics of Corrosion and Its Inhibition

**Figure 9:**
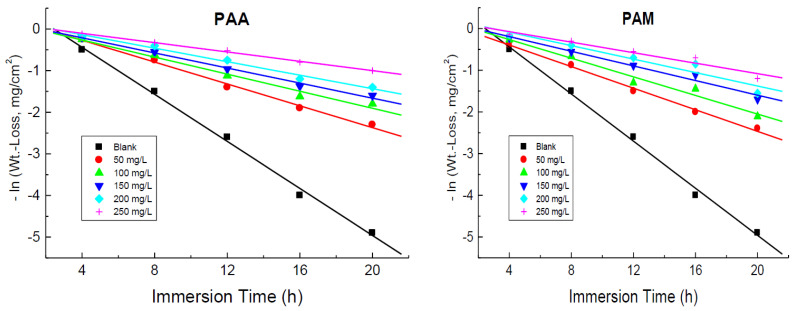
First-order rate constant plots in the corrosion of copper in 1.0 M HNO_3_ medium without and with addition of poly(acrylic acid) (PAA) and polyacrylamide (PAM) at 298 K.

The kinetics of copper corrosion in 1.0 M HNO_3_ medium and its inhibition by the tested polymers were investigated. The plots of –ln(weight loss) versus time were linear, as illustrated in Figure 9, indicating that the kinetics of copper corrosion in 1.0 M HNO_3_ medium and its inhibition by the tested polymers were negative first-order processes. The first-order rate constant values, *k*_1_ (in h^−1^), were evaluated from the slopes of these plots and are inserted in Table 4. The values of half-life times (*t*_1/2_, h) of this process were gained (Table 4) from the obtained values of *k*_1_ using Equation (9) [65]:

(9) $$t_{1/2}=\frac{0.693}{k_{1}}\;$$

**Figure 10:**
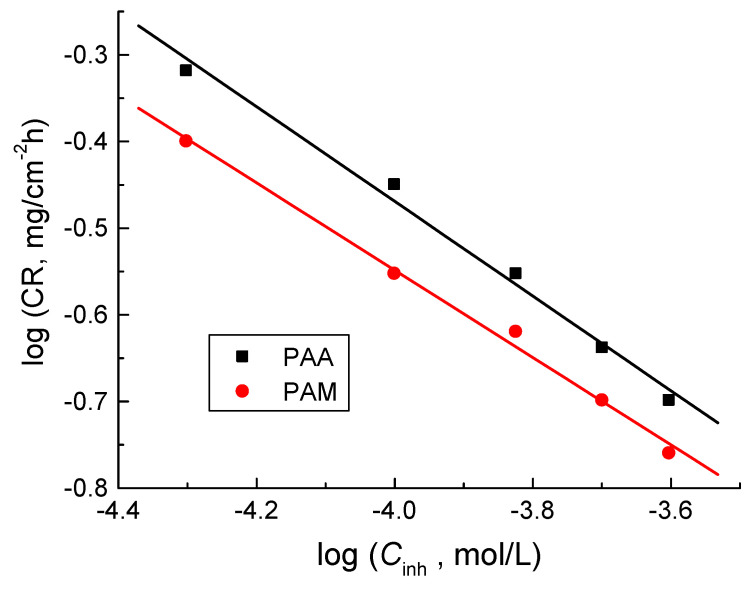
Log *CR* vs. log *C*_inh_ for the inhibition of copper corrosion in 1.0 M HNO_3_ medium and with addition of poly(acrylic acid) (PAA) and polyacrylamide (PAM) at 298 K.

Moreover, the values of the order (*n*) of corrosion inhibition were calculated using Equation (10) [66]:

log *CR* = log *k* + *n* log *C_inh_* (10)

where *k* is the specific rate constant (in mg/cm^2^ h). The plots of log *CR* versus log *C_inh_* for the tested polymers at 298 K were linear, as shown in Figure 10. Values of *n* were calculated from the slopes of such plots and were found to be −0.54 and −0.51 for poly(acrylic acid) and polyacrylamide, respectively. The acquired value of *n* suggested that the corrosion inhibition was a negative fractional first-order reaction regarding the polymers’ concentrations. The negative sign of *n* values as well as the opposite proportionality of the CRs with the polymers’ concentrations (Figure 10) refers to the good % IEs of the tested polymers [67].

### 3.2. PDP Measurements

**Figure 11:**
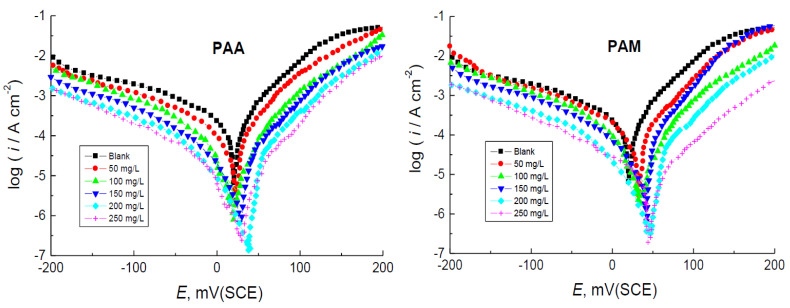
PDP curves (Tafel plots) for the corrosion of copper in 1.0 M HNO_3_ medium and with addition of poly(acrylic acid) (PAA) and polyacrylamide (PAM) at 298 K.

Figure 11 illustrates the Tafel plots for the corrosion of copper in 1.0 M HNO_3_ medium and in the presence of various concentrations (50–250 mg/L) of the tested polymers at 298 K. Values of the corrosion parameters gained from the Tafel plots, as corrosion potentials (*E*_corr_), anodic and cathodic gradients (*β*_a_, *β*_c_), corrosion current densities (*i*_corr_), polarization resistance (*R*_p_), and the values of both % IE and *θ* of the tested polymers, were calculated and are listed in Table 5. Figure 11 shows that addition of the tested polymers to 1.0 M HNO_3_ medium shifted the anodic and cathodic Tafel branches of PDP curves to less *i*_corr_ values. This performance led to hindering both anodic and cathodic reactions and thus inhibited the corrosion of copper. The value of *E*_corr_ for copper in the corrosive medium was slightly shifted towards positive or anodic direction upon addition of the tested polymers, suggesting that such polymers are mixed-kind inhibitors with anodic priority [68]. Furthermore, both values of *β*_a_ and *β*_c_ were decreased with the addition of the tested polymers, indicating that addition of these polymers hindered both the anodic dissolution of copper and the cathodic hydrogen evolution reactions, respectively, confirming that these polymers acted as mixed-kind inhibitors. The value of *i*_corr_ of copper in the corrosive medium was reduced, while that of *R*_p_ was increased with the increase of the polymers’ concentrations, indicating corrosion inhibition. The gained % IE values were increased with increasing polymers’ concentrations in the corrosive medium, and the trend of the obtained values of % IEs was polyacrylamide > poly(acrylic acid) in accordance with the obtained WL results.

### 3.3. EIS Measurements

**Figure 12:**
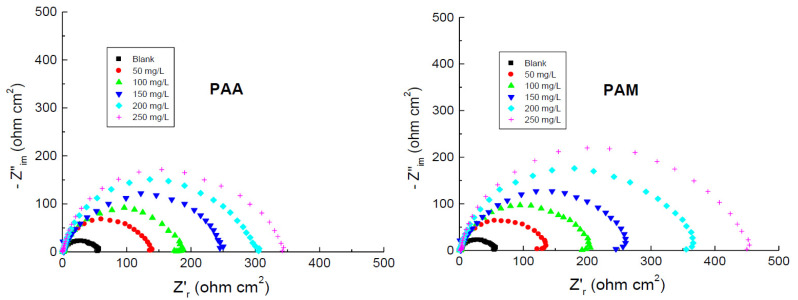
Nyquist plots for the corrosion of copper in 1.0 M HNO_3_ medium and with addition of poly(acrylic acid) (PAA) and polyacrylamide (PAM) at 298 K.

**Figure 13:**
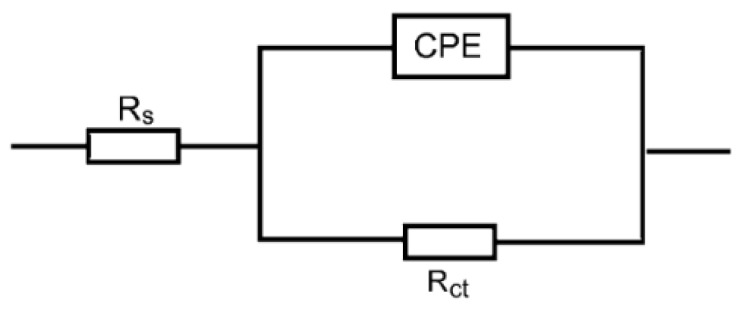
Electrochemical equivalent circuit utilized to fit the EIS output data for copper in 1.0 M HNO_3_ medium and with addition of the tested polymers.

The Nyquist plots for copper corrosion in 1.0 M HNO_3_ medium and with the addition of various concentrations of the tested polymers at 298 K are illustrated in Figure 12. The figure shows that the Nyquist plots of copper in the corrosive medium and with numerous concentrations of the tested polymers manifested single, depressed capacitive loops and one-time constants, indicating that the corrosion of copper was controlled by a charge-transfer process [69]. On the other hand, the acquired impedance spectra were analyzed by matching to the equivalent circuit, shown in Figure 13.

EIS parameters, namely *R*_s_ (solution resistance), *R*_ct_ (charge-transfer resistance), and CPE (constant phase element), were determined from the EIS spectra and are listed in Table 6. The % IE values and *θ* of the tested polymers were computed and are also inserted in Table 6. The calculated parameters elucidated that the value of *R*_ct_ for copper in the corrosive medium was increased with increasing polymers’ concentrations, with a decrease in the CPE value proving that the addition of the tested polymers reduced the corrosion rate of copper. Further, decreasing the value of CPE implies adsorption of the polymeric molecules on the copper/electrolyte interface [70], shielding the copper surface from the corrosive medium and thus enhancing the values of % IE.

**Figure 14:**
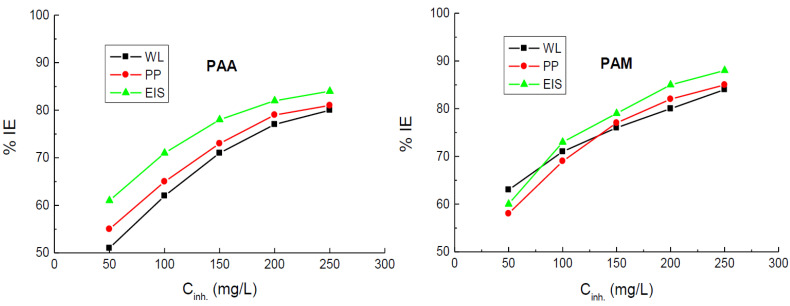
Comparing between the three employed techniques, i.e., WL, PDP, and EIS, for the change of the % IEs of poly(acrylic acid) (PAA) and polyacrylamide (PAM) with their concentrations for the corrosion of copper in 1.0 M HNO_3_ medium at 298 K.

Finally, the data listed in Table 1, Table 5 and Table 6 revealed a good agreement between all utilized techniques (WL, PDP, and EIS) regarding the values of % IEs at 298 K, as illustrated in Figure 14, indicating the validity of the employed measurements. In addition, from the obtained values of % IEs, it can be concluded that polyacrylamide is more efficient than poly(acrylic acid). This can be attributed to the difference in the chemical structures of both tested polymers, where polyacrylamide molecule contains O and N heteroatoms, while the poly(acrylic acid) molecule contains only two O atoms. Because of the existence of a lone pair of electrons and the difference in heteroatoms’ electronegativities, the inhibition efficiencies of molecules containing heteroatoms follows the order N > O [71], confirming the above-mentioned trend.

### 3.4. Surface Examination

**Figure 15:**
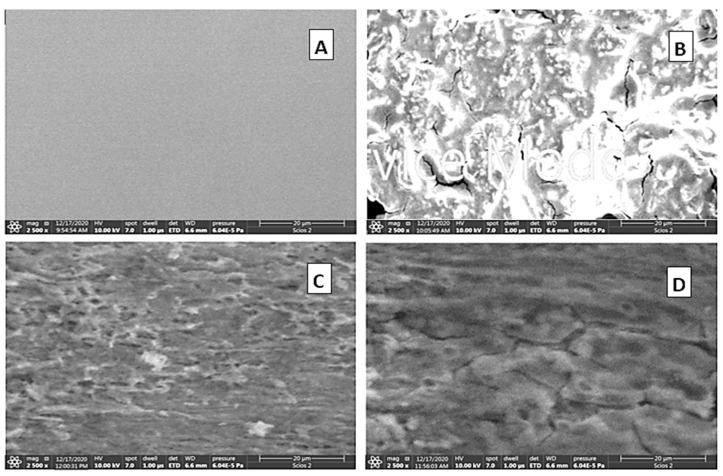
SEM images (mag. ×2500) of the surfaces of copper sheets: (**A**) before immersion, (**B**) after immersion in 1.0 M HNO_3_ medium for 12 h, and (**C**,**D**) after 12 h immersion in 1.0 M HNO_3_ with 250 mg/L of poly(acrylic acid) and polyacrylamide, respectively.

SEM micrographs of the copper surfaces in the examined corrosive medium (1.0 M HNO_3_) without and with adding a 250 mg/L of the tested polymers are illustrated in Figure 15A–D. The figure manifests a polished copper surface: (A) before and (B) after 12 h immersion in the corrosive medium. Figure 15B shows an excessive damage of the copper surface as a result of the aggressiveness of the corrosive medium. Figure 15C,D manifests SEM images after addition of 250 mg/L of the tested polymers to the corrosive medium. These two images show vanishing damage, which was observed on the copper surfaces in the polymer-free corrosive medium. This can be ascribed to the strong adsorption of polymeric molecules on the copper surface, leading to its protection.

### 3.5. Theoretical Studies

#### 3.5.1. Quantum Chemical Calculations

**Figure 16:**
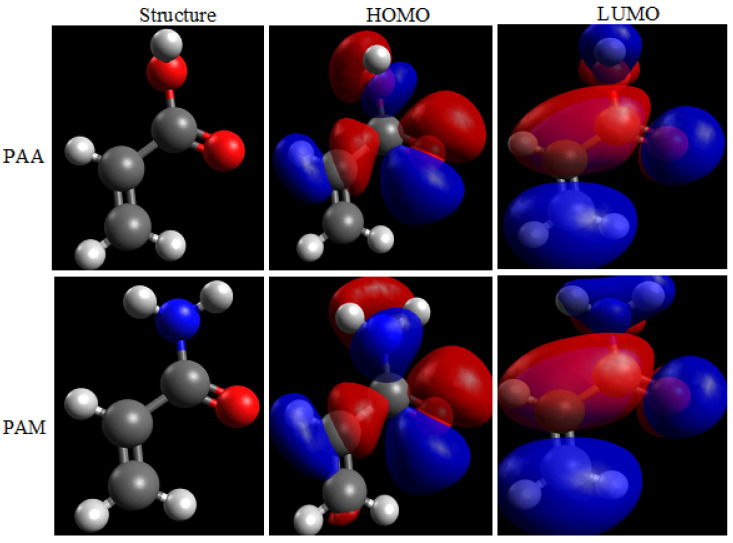
The obtained molecular structures, HOMO and LUMO, of the neutral inhibitor molecules by DFT/B3LYP/6-31G*(d,p).

With the substitution of two hydroxyl groups (OH) in PAA with amino groups (NH_2_) in PAM, the average inhibitory efficiency increased from 82% to 86% at 250 mg/L of inhibitor concentration, demonstrating the importance of the inhibitors’ molecular structure in adsorption. As a result, quantum chemical simulations were used to look into the structural factors that affect the effectiveness of inhibitor inhibition. Figure 16 displays the optimized chemical structures representative of one repeating unit of PAA and PAM polymers particles, with the lowest energy determined by DFT calculations. As a result, the calculated quantum parameters are only related to one polymer unit. The PAA polymer particles have a molecular weight of 45,000 g/mol and *n* = 625 repeating units. At the same time, the PAM polymer particles have a molecular weight of 40,000 g/mol and *n* = 563 repeating units.

Table 7 lists the quantum chemical factors that come from calculations that affect the effectiveness of inhibitor inhibition. The energies of the inhibitor molecule’s orbitals are related to the ionization potential (*I*) and electron affinity (*A*), according to Koopman’s theorem [72,73], where *I* = – *E*_HOMO_, and A = – *E*_LUMO_. Absolute electronegativity, *χ*, and absolute hardness, *η*, of the inhibitor molecule are given by *χ* = 0.5 × (*I* + *A*) and *η* = 0.5 × (*I* – *A*). Hardness is the opposite of softness: *σ* = 1/*η*. The fraction of the electron transported (Δ*N*) from the inhibitor to metallic surface is calculated using the values of *χ* and *η* as follows [74,75]: Δ*N* = 0.5 × (*χ*_Cu_ – *χ*_inh_)/(*η*_Cu_ + *η*_inh_). Pearson’s electronegativity scale is used, with theoretical values of *χ*_Cu_ = 4.48 eV/mol and *η*_Cu_ = 0 eV/mol for copper.

The electron-donating capacity of the *E*_HOMO_ and the solidity of contact with the metal substrate are well-known to increase with *E*_HOMO_ value. Similarly, the smaller the *E*_LUMO_ is, the tougher the acceptability of accepting electrons is, and the more solid the adsorption at the metal substrate is [76,77]. As a result, a smaller value of the energy gap Δ*E* correlates with a higher adsorption volume, which is advantageous for improving corrosion inhibition effectiveness. Table 7 shows that the Δ*E* value of PAA is 0.1990 eV, which is close to the value of PAM (0.2118 eV), indicating that their activities are not significantly different [78,79]. When the metal substrates and inhibitor particles are close to each other, the calculation shows that electrons will flow from the lower-electronegativity site to the higher-electronegativity site until equilibrium is reached. When the electron-transfer-fraction (Δ*N*) value is positive, electrons move from the inhibitor particles into the metal atoms on the copper substrate; when the Δ*N* value is negative, electrons move from the metal atoms on the copper substrate into the inhibitor particles, a process known as back-donation.

According to Table 7, both PAA and PAM have an exceptional ability to release electrons from the inhibitor’s particles to the Cu surface via co-ordination bonds, creating an effective protective film and preventing metal dissolution. This is shown by their positive electron-transfer fractions (Δ*N*), which show their exceptional ability to do so. One of the requirements for theoretical principles in corrosion investigation is the hard-soft-acid-base (HSAB) technique. While PAA and PAM are soft alkalis, copper might be categorized as a soft acid [80,81,82,83,84]. The combination of PAA, PAM, and Cu surface complies with the HSAB idea, as demonstrated in Table 7, where the relatively high global softness (*σ* = 1/*η*) of inhibitors indicates further information about the outstanding corrosion inhibitor.

#### 3.5.2. Molecular Dynamic Simulations

**Figure 17:**
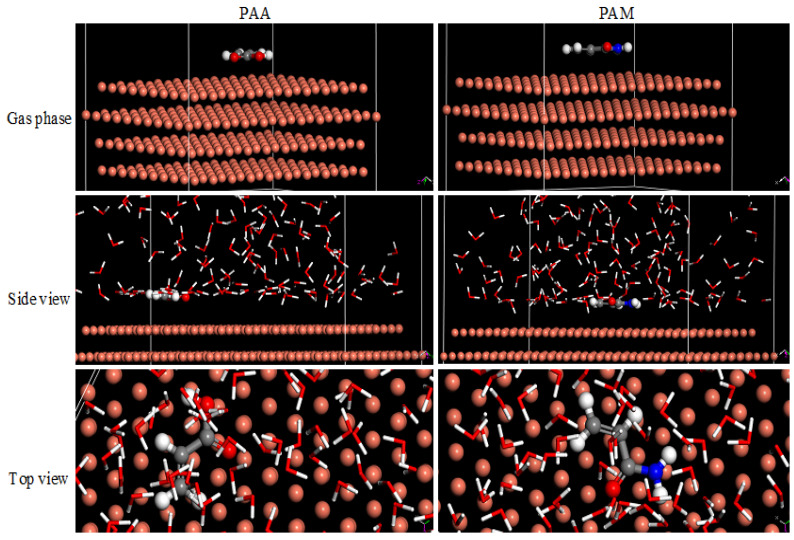
The adsorption density of PAA and PAM on copper (1 1 1) surface.

In order to well comprehend the communication between the investigated inhibitor particles and the Cu substrate, MD simulations have been made on Cu(111) substrate [85]. The equipoise outlines the repeating unit of PAA and PAM inhibitors particles at the copper via the side and top sights, exhibited in Figure 17. Both PAA and PAM conformers were firmly absorbed at the Cu(111) substrate in a matching manner. Equated to further adsorption instructions, in order to suspend metallic deterioration, it is sensible that the thoroughgoing handle is produced in a parallel way. Based on the investigation of contacting mechanism, it inspires electron at the inhibitor particles move to the vacant orbitals of surface copper atoms, which form a defensive layer [86]. The adsorptive energy could facilitate the binding permanency between the inhibitor particles and Cu substrate. The interaction energies (*E*_interaction_) and binding energies (*E*_binding_) computed are useful to clarify the interactions between the considered inhibitor particles at the Cu substrate in the reproduction system, which can be defined in the following Equations:

(11) $$E_{\mathrm{interaction}}=E_{\mathrm{total}}-\left(E_{\mathrm{surface}+H_{2}O}+E_{\mathrm{inhibitor}}\right)\;$$

(12) $$E_{\mathrm{binding}}=-E_{\mathrm{interaction}}$$

The total energy (*E*_total_) denotes the totality energy of the H_2_O particles, the considered inhibitor particles, and the Cu surface. In addition, the value of $E_{\mathrm{surface}+H_{2}O}$ corresponds to the energy of the Cu surface and acid solution. In balanced situations, the *E*_binding_ demands that the unit of PAM (–152.374 kcal/mol) > unit of PAA (–133.652 kcal/mol). Therefore, the maximum value of *E*_binding_ was obtained for PAM particles, signifying that it has a tougher and more impulsive adsorptive behavior at the Cu substrate than PAA particles and hence has an advanced percentage inhibition efficiency of corrosion.

### 3.6. Proposed Corrosion Inhibition Mechanisms of Copper in Nitric Acid Solution

The corrosion of Cu in HNO_3_ solution was explained in an earlier study as follows [87]:

Anodic site:

Cu → Cu^2+^ + 2e^−^ (13)

Cathodic site:

O_2_ + 4H^+^ + 4e^−^ → 2H_2_O (14)

Alongside this, NO_3_^−^ ions are quickly reduced, as indicated by the following equations:

NO_3_**^−^** + 4H^+^ + 3e^−^ → NO + 2H_2_O (15)

NO_3_^−^ + 3H^+^ + 2e^−^ → HNO_2_ + H_2_O (16)

As shown in Equation (11), copper metal is corroded and oxidized to Cu^2+^. It was reported that copper can form various oxide phases on its surface, which somewhat protect it. However, in HNO_3_ media, no oxide film is constructed to protect the surface from corrosion as a result of the following reactions [88]:

Cu_2_O + 2H^+^ ⇌ 2Cu^+^ + H_2_O (17)

CuO + 2H^+^ ⇌ Cu^2+^ + H_2_O (18)

Furthermore, Cu^+^ ions are disproportionated via the reaction [89]:

2Cu^+^ ⇌ Cu^2+^ + Cu (19)

The higher IEs value obtained with the screened polymers can be attributed to their strong adsorption on the Cu surface. This is due to the fact that these compounds contain lone pairs of electrons on the heterocyclic atoms present in their chemical structures (O atoms in poly (acrylic acid) and O and N atoms in polyacrylamide) that can form coordination bonds with the vacant d-orbital of copper, resulting in construction of a protective layer(s).

By examining the data obtained from this study, it can be seen that the E_corr_ value of Cu in the 1.0 M HNO_3_ solution was positive, and thus, the Cu-surface becomes positively charged. Thus, negatively charged nitrate ions (NO_3_**^−^**) can easily and quickly chemically adsorb on the surface of the copper and turn its surface into a negatively charged one.

Meanwhile, the tested polymers, because they contain polar electron-donating atoms (i.e., O, and N), can be protonated in the acidic solution to form positively charged complex ions, as shown in the equation:

Polymer + nH^+^ ⇌ [Polymer − Hn] ^n+^ (20)

Accordingly, electrostatic attraction is expected to occur between the positively charged polymer particles and the negatively charged copper surface, leading to the construction of a highly adsorbed (physisorption) layer that protects the copper surface from the attack of the corrosive medium. Indeed, this is not the only expected adsorption site between the polymer and the copper surface, as polymeric molecules and their protonated species can adsorb on the anodic and cathodic sites, respectively. Adsorption may occur on the anodic sites through the O and N atoms of the polymer, causing a retardation in the dissolution of Cu, while the adsorption on the cathodic sites through the protonated polymer may occur, which hinders the oxygen evolution reaction. As an alternative possibility, the formation of donor–acceptor complexes (precipitates) on the metal surface between the inhibitor lone pairs and the vacant d-orbital of the metal has been reported to be the cause of corrosion inhibition [89]. Hence, we conclude that the corrosion of copper in a nitric acid solution may be inhibited by one or both of the following two processes: the formation of a protective layer (s) and/or the formation of a precipitate on the surface of the metal.

## 4. Conclusions

1. Cu corrosion in 1.0 M HNO_3_ medium and its inhibition using poly(acrylic acid) and polyacrylamide polymers were investigated using various tools.
2. The tested polymers were set to be efficient inhibitors for Cu corrosion in 1.0 M HNO_3_ medium, and the values of inhibition efficiencies of poly(acrylic acid) are slightly higher than those recorded for polyacrylamide.
3. Thermodynamic and kinetic parameters were determined that sustain the mechanism of physical adsorption of the tested polymers.
4. The proposed adsorption of the polymeric molecules on the copper surface obeyed the Langmuir isotherm.
5. The tested polymers were found to perform as mixed-type inhibitors with anodic priority.
6. The kinetics and mechanisms of copper corrosion and its inhibition were investigated.
7. There is a good agreement between all employed techniques.
8. The creation of connections between inhibitors’ active sites and copper atoms was predicted by DFT simulations, and their increased affinity for metal surface was supported by their tight and parallel distribution over its surface. The chemicals used in this investigation demonstrated to be potential corrosion inhibitors.

## Figures and Tables

**Table 1:** Values of *CR* of copper in 1.0 M HNO_3_ medium, % IE, and *θ* of numerous concentrations of poly(acrylic acid) (PAA) and polyacrylamide (PAM) at different temperatures.

Polymer	Conc. (mg/L)	Temperature (K)											
		288			298			308			318		
		*CR*	% IE	*θ*	*CR*	% IE	*θ*	*CR*	% IE	*θ*	*CR*	% IE	*θ*
Blank	0	75	--	--	98	--	--	112	--	--	123	--	--
PAA	50	36	58	0.58	48	51	0.51	60	46	0.46	68	45	0.45
	100	22	71	0.71	37	62	0.62	47	58	0.58	57	54	0.54
	150	16	79	0.79	28	71	0.71	36	68	0.68	43	65	0.65
	200	12	84	0.84	23	77	0.77	30	73	0.73	37	70	0.70
	250	11	85	0.85	20	80	0.80	26	77	0.77	34	72	0.72
PAM	50	23	69	0.69	36	63	0.63	49	56	0.56	63	49	0.49
	100	15	80	0.80	28	71	0.71	38	66	0.66	48	61	0.61
	150	10	87	0.87	24	76	0.76	30	73	0.73	37	70	0.70
	200	7	91	0.91	20	80	0.80	24	79	0.79	31	75	0.75
	250	5	93	0.93	16	84	0.84	22	80	0.80	27	78	0.78

**Table 2:** Values of *K*_ads_ and thermodynamic parameters for the corrosion of copper in 1.0 M HNO_3_ medium in the presence of poly(acrylic acid) (PAA) and polyacrylamide (PAM) at different temperatures.

Polymer	Temp. (K)	10^−3^ *K_ads_* L mol^−1^	$\Delta G_{ads}^{o}$ kJ mol^−1^	$\Delta H_{ads}^{o}$ kJ mol^−1^	$\Delta S_{ads}^{o}$ J mol^−1^ K^−1^
PAA	288	1.69	−27.42	−13.42	48.61
	298	1.22	−27.56		47.45
	308	1.02	−28.03		47.44
	318	0.92	−28.66		47.92
PAM	288	2.48	−28.33	−17.16	38.78
	298	1.88	−28.63		38.49
	308	1.51	−29.03		38.54
	318	1.26	−29.49		38.77

**Table 3:** Values of the activation parameters for the corrosion of copper in 1.0 M HNO_3_ medium and with addition of poly(acrylic acid) (PAA) and polyacrylamide (PAM).

Polymer	Drug Conc. (mg/L)	*E*_a_* kJ mol^−1^	∆H* kJ mol^−1^	∆S* J mol^−1^ K^−1^
Blank	0	12.39	9.81	−3.74
PAA	50	16.38	13.72	3.74
	100	19.12	19.04	19.13
	150	21.12	22.70	28.27
	200	23.53	26.11	37.83
	250	28.60	30.76	51.55
PAM	50	25.44	22.86	32.01
	100	27.43	24.94	36.17
	150	30.76	28.85	46.56
	200	34.92	33.26	58.62
	250	41.32	40.41	80.24

**Table 4:** Values of the first-order rate constant (*k*_1_) and half-life time (*t*_1/2_) for the corrosion of copper in 1.0 M HNO_3_ medium and with addition of poly(acrylic acid) (PAA) and polyacrylamide (PAM) at 298 K.

Polymer Conc. (mg/L)	PAA		PAM	
	10^3^ *k*_1_, h^−1^	*t*_1/2_, h	10^3^ *k*_1_, h^−1^	*t*_1/2_, h
Blank	282	2.46	282	2.46
50	131	5.29	129	5.37
100	100	6.93	112	6.19
150	90	7.70	86	8.06
200	82	8.451	80	8.66
250	62	11.18	65	10.66

**Table 5:** Corrosion parameters for the corrosion of copper in 1.0 M HNO_3_ medium and with addition of poly(acrylic acid) (PAA) and polyacrylamide (PAM) at 298 K.

Polymer	Conc. (mg/L)	*E*_corr_ (mV(SCE))	*β*_a_ (mV/dec.)	*−β*_c_ (mV/dec.)	*i*_corr_ (µA/cm^2^)	*R*_p_ (ohm cm^2^)	% IE	*θ*
Blank	0	22	56	121	211	79	--	--
PAA	50	24	50	111	95	158	55	0.55
	100	24	52	96	74	200	65	0.65
	150	27	47	94	57	239	73	0.73
	200	36	45	91	44	298	79	0.79
	250	28	42	96	40	318	81	0.81
PAM	50	31	46	97	89	152	58	0.58
	100	37	49	99	65	219	69	0.69
	150	32	38	95	49	241	77	0.77
	200	42	45	102	38	357	82	0.82
	250	48	55	106	32	492	85	0.85

**Table 6:** Values of impedance parameters for the corrosion of copper in 1.0 M HNO_3_ medium and with addition of poly(acrylic acid) (PAA) and polyacrylamide (PAM) at 298 K.

Polymer	Conc. (mg/L)	*R*_s_ (ohm cm^2^)	*R*_ct_ (ohm cm^2^)	CPE (µF/cm^2^)	% IE	*θ*
Blank	0	1.2	55	288	--	--
PAA	50	1.3	141	112	61	0.61
	100	1.7	190	98	71	0.71
	150	1.9	250	83	78	0.78
	200	2.7	305	66	82	0.82
	250	1.5	344	57	84	0.84
PAM	50	1.8	138	113	60	0.60
	100	1.6	204	95	73	0.73
	150	2.1	262	79	79	0.79
	200	1.5	367	54	85	0.85
	250	1.7	458	43	88	0.88

**Table 7:** Quantum chemical parameters derived for PAA and PAM calculated with the DFT method.

Parameters	PAA	PAM
*E*_HOMO_	−0.2645	−0.2708
*E*_LUMO_	−0.0527	−0.0718
Δ*E*_L-H_	0.2118	0.1990
*I*	0.2645	0.2708
*A*	0.0527	0.0718
*χ*	0.1586	0.1713
*η*	0.1059	0.0995
*σ*	9.4429	10.0503
Δ*N*	20.6520	22.0126
